# Loosenin-Like Proteins from *Phanerochaete carnosa* Impact Both Cellulose and Chitin Fiber Networks

**DOI:** 10.1128/aem.01863-22

**Published:** 2023-01-16

**Authors:** Mareike Monschein, Eleni Ioannou, Taru Koitto, Leamon A. K. M. Al Amin, Jutta J. Varis, Edward R. Wagner, Kirsi S. Mikkonen, Daniel J. Cosgrove, Emma R. Master

**Affiliations:** a Department of Bioproducts and Biosystems, Aalto University, Espoo, Finland; b Department of Food and Nutrition, University of Helsinki, Helsinki, Finland; c Department of Biology, Pennsylvania State University, University Park, State College, Pennsylvania, USA; d Center for Lignocellulose Structure and Formation, Pennsylvania State University, University Park, State College, Pennsylvania, USA; e Department of Chemical Engineering and Applied Chemistry, University of Toronto, Toronto, Ontario, Canada; Chalmers University of Technology

**Keywords:** loosenin, expansin, *Phanerochaete carnosa*, cellulose, chitin, biotechnology, carbohydrate active enzymes, carbohydrate binding, filamentous fungi, lignocellulose

## Abstract

Microbial expansin-related proteins are ubiquitous across bacterial and fungal organisms and reportedly play a role in the modification and deconstruction of cell wall polysaccharides, including lignocellulose. So far, very few microbial expansin-related proteins, including loosenins and loosenin-like (LOOL) proteins, have been functionally characterized. Herein, four LOOLs encoded by Phanerochaete carnosa and belonging to different subfamilies (i.e., PcaLOOL7 and PcaLOOL9 from subfamily A and PcaLOOL2 and PcaLOOL12 from subfamily B) were recombinantly produced and the purified proteins were characterized using diverse cellulose and chitin substrates. The purified PcaLOOLs weakened cellulose filter paper and cellulose nanofibril networks (CNF); however, none significantly boosted cellulase activity on the selected cellulose substrates (Avicel and Whatman paper). Although fusing the family 63 carbohydrate-binding module (CBM63) of BsEXLX1 encoded by Bacillus subtilis to PcaLOOLs increased their binding to cellulose, the CBM63 fusion appeared to reduce the cellulose filter paper weakening observed using wild-type proteins. Binding of PcaLOOLs to alpha-chitin was considerably higher than that to cellulose (Avicel) and was pH dependent, with the highest binding at pH 5.0. Amendment of certain PcaLOOLs in fungal liquid cultivations also impacted the density of the cultivated mycelia. The present study reveals the potential of fungal expansin-related proteins to impact both cellulose and chitin networks and points to a possible biological role in fungal cell wall processing.

**IMPORTANCE** The present study deepens investigations of microbial expansin-related proteins and their applied significance by (i) reporting a detailed comparison of diverse loosenins encoded by the same organism, (ii) considering both cellulosic and chitin-containing materials as targeted substrates, and (iii) investigating the impact of the C-terminal carbohydrate binding module (CBM) present in other expansin-related proteins on loosenin function. By revealing the potential of fungal loosenins to impact both cellulose and chitin-containing networks, our study reveals a possible biological and applied role of loosenins in fungal cell wall processing.

## INTRODUCTION

Expansins were first identified through studies of acid-induced growth of cucumber hypocotyls (Cucumis sativus), where they trigger cell wall loosening without evidence of lytic activity (1–3). Plant expansins have since been implicated in fruit ripening, root hair elongation, germination, pollen tube penetration, and other developmental processes (4, 5). Although their mode of action remains elusive, detailed biochemical and biophysical studies indicate that expansins disrupt noncovalent bonds at so-called “biomechanical hot spots,” which are load-bearing junctions between cellulose microfibrils or between cellulose microfibrils and other matrix polymers (6–8). Expansins have been identified across land plants (4, 5, 9, 10), and the ubiquity of expansin-related proteins among microorganisms has since been verified through genome sequencing (9, 11–14).

All expansins exhibit a distinctive two-domain structure of 250 to 275 amino acids. The N-terminal domain (D1) is a six-stranded double-psi beta-barrel (DPBB) which is packed tightly next to a C-terminal domain (D2) having a β-sandwich fold (15). D1 and D2 domains align to form a long, shallow groove with highly conserved polar and aromatic residues suitable to bind a twisted polysaccharide chain (15–17). D1 is structurally related to the catalytic domain of family 45 glycoside hydrolases (GH45); however, it lacks the aspartate that serves as the catalytic base in GH45 enzymes (16), and so far, no lytic activity has been observed for any plant expansin or bacterial expansin-like protein (10). D2 is homologous to group 2 grass pollen allergens and has been classified as a family 63 carbohydrate binding module (CBM63) (17).

Expansins and expansin-related proteins are classified based on phylogenetic analysis (13, 14, 18). Plant-derived expansins are classified as α-expansins (EXPA), β-expansins (EXPB), and two small groups of expansin-like sequences (EXLA and EXLB). Microbial expansins (EXLX) include proteins with both D1 and D2 domains. Some expansin-related proteins from microbes possess other domain architectures, including proteins comprising only the D1 domain (e.g., loosenins [19] and cerato-platanins [20]) and multidomain proteins comprising other domains in addition to D1 and D2 (e.g., swollenins [16, 21, 22]). So far, most studies of microbial expansin-related proteins have focused on their potential to boost enzymatic hydrolysis of lignocellulosic substrates to sugars (23–26). Reported impacts on lignocellulose deconstruction, however, vary and depend strongly on the biomass source (27).

In an effort to deepen our understanding of the molecular function and applied potential of microbial expansin-related proteins, we recombinantly produced and characterized four loosenin-like proteins (LOOLs) encoded by Phanerochaete carnosa and expressed during growth on wood substrates (28, 29). Only two loosenins have been previously characterized: LOOS1 from the white-rot basidiomycete Bjerkandera adusta (19) and N2 from Neurospora crassa (30). Both LOOS1 and N2 reportedly disrupt the structure of cotton fibers (19, 30); pretreatment of Agave tequilana fibers with LOOS1 also increases the susceptibility of the material toward enzymatic hydrolysis, enhancing the rate of reducing sugar release by up to 7.5 times (19). In addition to the functional characterization of wild-type LOOLs from *P. carnosa* (PcaLOOLs), C-terminal fusions were constructed herein to investigate the impact of appending a D2 domain on the action of loosenin-like proteins.

## RESULTS AND DISCUSSION

### Sequence analysis.

Previous transcriptomic analysis of *P. carnosa* grown on aspen or spruce uncovered the expression of 12 LOOLs, which were classified into phylogenetic subgroup A and subgroup B (28, 29). Whereas transcript abundances for PcaLOOL12 were comparatively high during *P. carnosa* cultivation on wood, PcaLOOL2 transcript abundances were highest overall (28, 29). PcaLOOL2 and PcaLOOL12 belong to subgroup B and are 25% and 34% identical to LOOS1 from *B. adusta*, respectively (28). In addition to the selection of PcaLOOL2 and PcaLOOL12 for recombinant expression and characterization, PcaLOOL7 and PcaLOOL9 were selected from subgroup A and are 64% and 59% identical to LOOS1, respectively.

**FIG 1:**
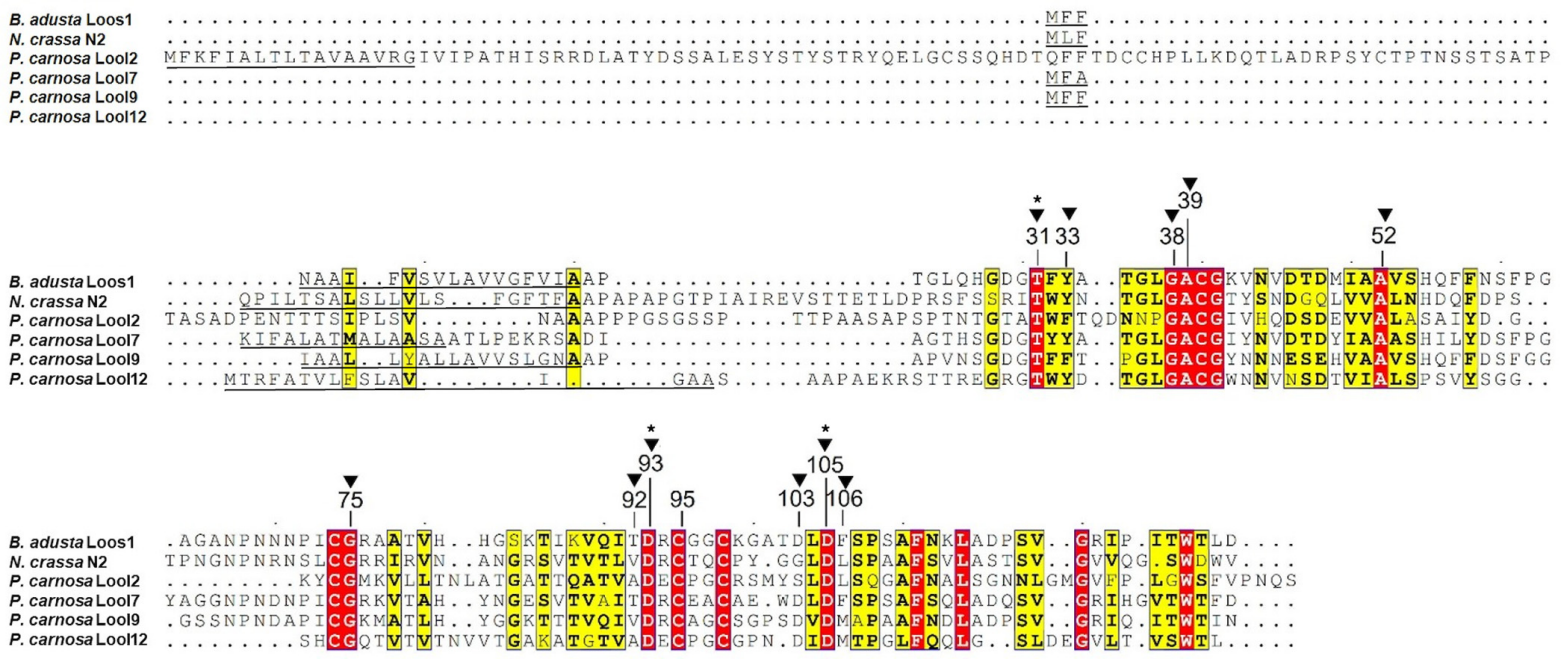
Multiple amino acid sequence alignment of *P. carnosa* LOOLs with characterized loosenins *B. adusta* LOOS1 and N. crassa N2. Strictly conserved regions are indicated by red blocks, and similar residues are indicated by yellow blocks. Gray boxes indicate chemical similarity across a group of residues. Numbering of amino acid residues corresponds to positions in LOOS1 and includes the predicted N-terminal signal sequence (underlined). Triangles indicate residues contributing to form a shallow binding groove or hydrogen bonds. Stars indicate residues proposed to be crucial for the activity of EXLX1. Amino acid sequences of *P. carnosa* PcaLOOL2 (GenBank accession no. EKM55357.1), PcaLOOL7 (GenBank accession no. EKM53490.1), PcaLOOL9 (GenBank accession no. EKM52742.1), PcaLOOL12 (GenBank accession no. EKM51974.1), N. crassa N2 (GenBank accession no. XP_959591.1), and *B. adusta* Loos1 (GenBank accession no. ADI72050.2) were retrieved from the NCBI protein database. Alignments were performed with Clustal Omega, and the figure was generated with ESPript 3.

The microbial expansin EXLX1 encoded by Bacillus subtilis (BsEXLX1) comprises a polysaccharide binding surface (PBS) lined with aromatic and polar residues that span the D1 and D2 domains of the protein (15, 31). Several of the D1 residues that form the PBS in BsEXLX1 are conserved in loosenins, including Gly38, Ala39, Ala52, and Asp93 of LOOS1 (Fig. 1; numbering of amino acids includes the N-terminal secretion signal). Notably, PcaLOOL2 and PcaLOOL12, as well as the N2 loosenin, harbor an insertion upstream of the PBS. PcaLOOL2 and PcaLOOL12 are further distinguished by an 8- to 9-amino acid deletion between residues equivalent to Gly63 and Pro72 in LOOS1.

Thr12 and Asp82 in BsEXLX1 (previously reported numbering; does not include the N-terminal secretion signal) are predicted to form hydrogen bonds to glucan molecules and are strictly conserved among all microbial expansin-related proteins, including the selected PcaLOOLs (Fig. 1). An alanine substitution for Asp82 resulted in a complete loss of BsEXLX1 activity, whereas an alanine substitution for Thr12 reduced BsEXLX1 activity by 70% (17); accordingly, the Asp82-Thr12 pair constitutes the presumptive active site of the D1 domain (12, 24). Asp82 in BsEXLX1 corresponds to the sole catalytic residue of lytic transglycosylases like Escherichia coli MltA (EcMltA) and to the proton donor in the catalytic site of GH45 enzymes. Thr12 in BsEXLX1 is equivalent to the Thr residue in the TWY motif of EXPA and the TFY motif of EXPB plant expansins. The hydroxyl group of Thr12 participates in a conserved hydrogen bond with the carboxylic group of Asp82 and is conserved in the catalytic site of GH45 enzymes and possibly MltA enzymes, where it is likely important for the proper positioning of the catalytic Asp (9, 15).

Notable differences between loosenins (and loosenin-like proteins) and the D1 domain of BsEXLX1 include the replacement of Tyr97 in BsEXLX1 with a cysteine (e.g., Cys95 in LOOS1). Tyr97 in BsEXLX1 is important, but not essential, to cell wall creep (17). Three other Cys residues are conserved among loosenins, and sequence analyses with DISULFIND predict putative disulfide bond formation between positions 74 to 98 and 40 to 95. The N-terminal extension of PcaLOOL2 harbors four additional Cys residues that could form two additional disulfide bonds. While disulfide bridges are absent from BsEXLX1, plant expansins possess three disulfide bridges in D1, whose six participating Cys residues are well conserved among EXPA and EXPB families (9, 15, 16). EXLX1 from Schizophyllum commune (ScEXLX1) also contains two disulfide bridges in D1, and the involved residues align with Cys40, Cys74, Cys95, and Cys98 of loosenins (32) (see Fig. S1 in the supplemental material).

### Production of recombinant PcaLOOLs and PcaLOOL fusions.

Codon-optimized genes encoding PcaLOOL2, PcaLOOL7, PcaLOOL9, and PcaLOOL12 were obtained as subcloned in pPICZαA and heterologously expressed in Pichia pastoris SMD1168H under the control of a methanol-inducible promoter. The recombinant PcaLOOLs were secreted and purified by Ni-nitrilotriacetic acid (Ni-NTA) affinity chromatography; the yields of PcaLOOL2, PcaLOOL7, PcaLOOL9, and PcaLOOL12 were 22, 28, 9, and 27 mg/L, respectively. The electrophoretic molecular weights of PcaLOOL2 and PcaLOOL9 were higher than expected, which was attributed to glycosylation in both cases (Fig. S2). The identity of all protein bands was confirmed by matrix-assisted laser desorption ionization–time of flight mass spectrometry (MALDI-TOF MS) analysis (Fig. S2B and C), and circular dichroism (CD) spectroscopy indicated that all PcaLOOLs were well folded (Fig. S3A). Specifically, all PcaLOOLs showed similar CD spectra indicating β-sheet-rich structures, consistent with the predicted DPBB fold (Fig. S3B). The CD spectra of PcaLOOL2 and PcaLOOL7 also showed a positive peak at ~220 nm (Fig. S3A), which has been assigned to the poly-l-proline type II (polyproline-II or PPII) type of secondary structure (33). During thermal unfolding, all PcaLOOLs underwent a single thermal transition as a function of temperature.

To investigate the impact of a D2 expansin domain on the action of loosenin-like proteins, the characterized CBM63 from the BsEXLX1 expansin-like protein (15, 31) was appended to PcaLOOLs from both subgroups A and B. Whereas PcaLOOL7 was selected from subgroup A, both PcaLOOL2 and PcaLOOL12 were included from subgroup B because PcaLOOL2 is twice the size of PcaLOOL12. The recombinant PcaLOOL fusions were secreted and purified by Ni-NTA affinity chromatography (Fig. S4); the yields of PcaLOOL2-CBM63, PcaLOOL7-CBM63, and PcaLOOL12-CBM63 were 71, 37, and 16 mg/L, respectively. The corresponding CBM63 was recombinantly produced in Escherichia coli; the protein yield was 42 mg/L.

### Lytic activity.

The loosenins produced herein were tested for lytic activity, including hydrolytic activity. Consistent with previous analyses of expansin-related proteins, none of the PcaLOOLs produced hydrolysis or lytic products from any of the tested polysaccharides, including xylan, xyloglucan, glucomannan, β-glucan, peptidoglycan, carboxymethyl cellulose, cellohexaose, and hexacetyl-chitohexaose (Fig. S5). The absence of hydrolytic activity in expansins has been attributed to the absence of a key aspartic acid residue (Asp10 in the family 45 glycoside hydrolase from Humicola insolens, HiCel45A) that functions as the general base in many GH45 endoglucanases. In contrast, the catalytic proton donor (Asp121 in HiCel45A) is conserved in expansins and expansin-related proteins (Asp82 in BsEXLX1; Asp105 in LOOS1) (15, 17, 34). Molecular dynamic simulations of the HiCel45A D10N mutant indicated the possibility of a nonhydrolytic reaction mechanism when the catalytic base aspartic acid is missing, as in EcMtlA (35). EcMltA utilizes only one acidic amino acid to cleave the β-1,4-glycosidic bonds in bacterial cell wall peptidoglycan (36). Nevertheless, the apparent lack of lytic activity for PcaLOOLs is in line with extensive experimental evidence debunking the proposed lytic action of expansins (37).

### Activity of PcaLOOLs on cellulose substrates.

**FIG 2:**
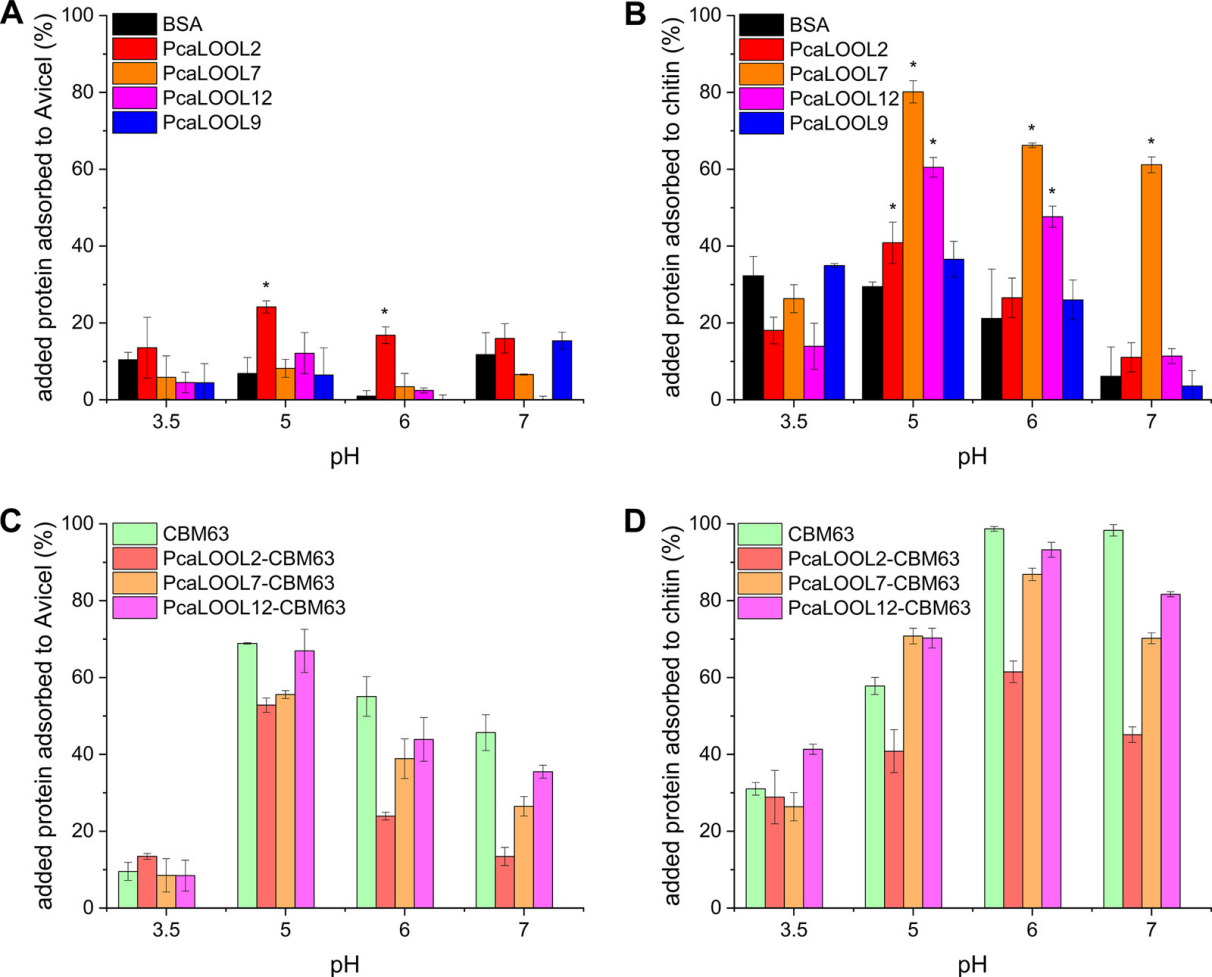
Adsorption of PcaLOOLs and PcaLOOL fusions to cellulose and chitin. The wild-type PcaLOOLs and fusion constructs (0.1 mg/mL) were incubated with 25 mg/mL substrate for 1 h at room temperature in a tube rotator set at 20 rpm. Unbound protein in the supernatant was determined by the BCA assay. The percentage of added protein adsorbed to substrate was calculated considering protein and substrate blanks. BSA was used as a reference. Asterisks indicate statistical significance (*P* ≤ 0.05; two-tailed *t* test) of increased PcaLOOL adsorption compared to that of BSA. (A) Adsorption of PcaLOOLs to Avicel PH-101 at pH 3.5, 5.0, 6.0, or 7.0; *n* = 3, error bars indicate standard deviations of the mean. (B) Adsorption of PcaLOOLs to chitin from shrimp shells at pH 3.5, 5.0, 6.0, or 7.0; *n* = 3, error bars indicate standard deviations of the mean. (C) Adsorption of the family 63 carbohydrate-binding module (CBM63) of BsEXLX1 and PcaLOOL-CBM63 fusions to Avicel PH-101 at pH 3.5, 5.0, 6.0, or 7.0; *n* = 3, error bars indicate standard deviations of the mean. (D) Adsorption of the CBM63 of BsEXLX1 and PcaLOOL-CBM63 fusions to chitin from shrimp shells at pH 3.5, 5.0, 6.0, or 7.0; *n* = 3, error bars indicate standard deviations of the mean.

**(i) Binding to cellulose.** Binding of the recombinant PcaLOOLs and PcaLOOL fusions to cellulose was measured as a first step to evaluating their potential nonlytic action on cellulosic materials (Fig. 2A). Considering the wild-type proteins, the highest levels of binding to cellulose were measured for PcaLOOL2 at pH 5.0 to pH 6.0, where between 20 and 25% of PcaLOOL2 adsorbed to the cellulose substrate (Avicel PH-101) after 1 h at room temperature. Similarly, Quiroz-Castañeda et al. (19) reported preferential adsorption of LOOS1 to Avicel PH-101 in comparison to that to bovine serum albumin (BSA). For all other PcaLOOLs, however, the extent of binding to Avicel PH-101 was negligible and indistinguishable from binding values obtained using BSA. Similarly, previous studies showed that the D1 domain of BsEXLX1 does not bind to Avicel (17). The higher binding of PcaLOOL2 to cellulose in comparison to that of the other PcaLOOLs was not explained by differences in surface potential calculated from model protein structures or the N-terminal extension of PcaLOOLs, which is predicted to include a helix-rich structure lacking similarity to any known protein domain (Fig. S2). Instead, the observed binding could be explained by the apparent high glycosylation of PcaLOOL2 (Fig. S2), given previous reports that show certain glycosylations can positively impact protein binding to crystalline cellulose (38). Fusing the CBM63 D2 domain of BsEXLX1 to PcaLOOL2, PcaLOOL7, and PcaLOOL12 clearly increased their binding to Avicel, particularly at pH 5.0, where the measured increases in binding were 1.2, 5.8, and 4.5 times, respectively (Fig. 2C).

**FIG 3:**
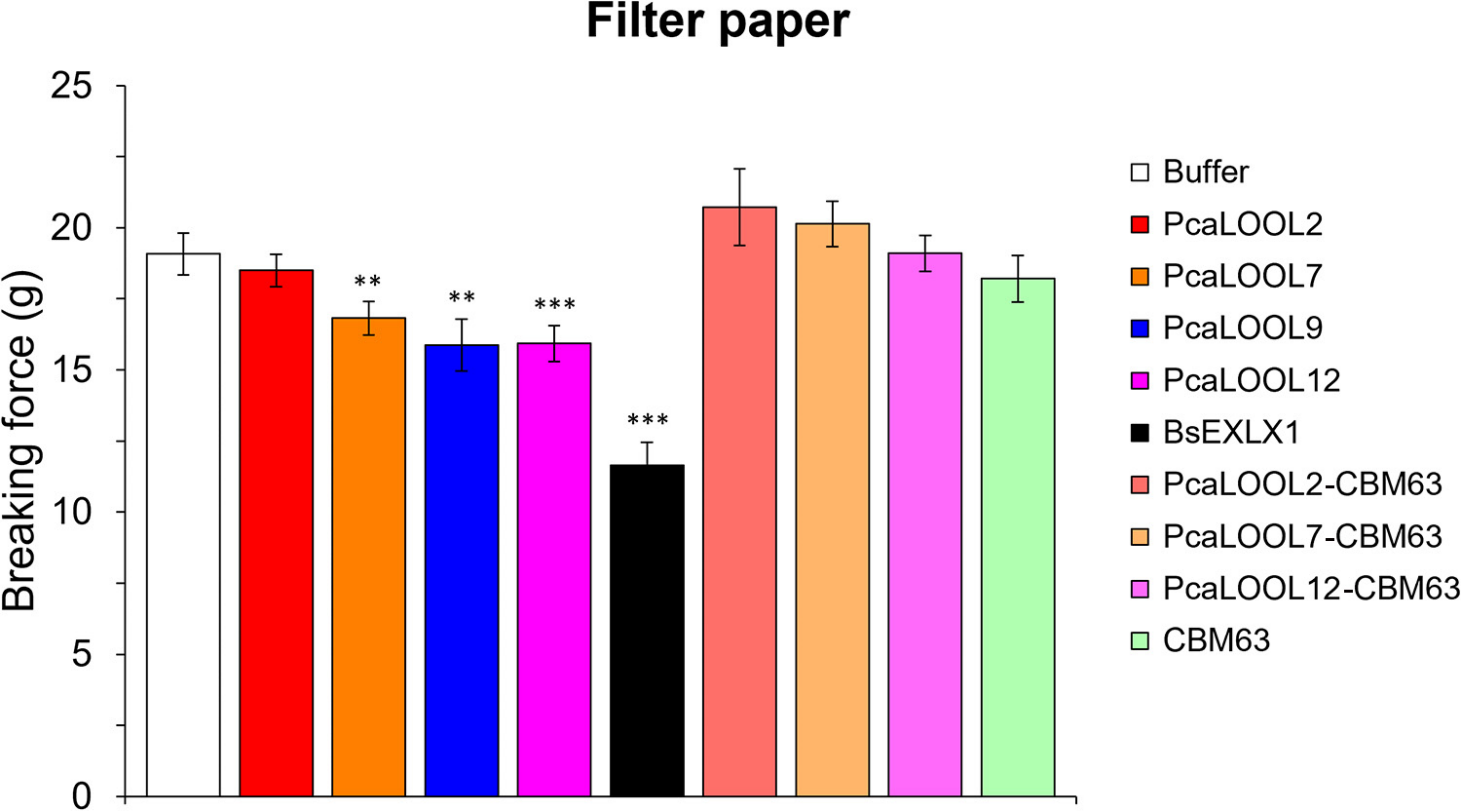
Breaking strength of filter paper after treatment with PcaLOOLs and fusion constructs. Strips of Whatman qualitative filter paper grade 3 were treated with or without 0.2 mg/mL protein in 20 mM MES buffer, pH 6, for 4 h at 25°C. The strips were extended at 1.5 mm/min, and the breaking force was recorded. BsEXLX1 was used as a positive reference. Error bars indicate ±standard errors of the mean, with *n* = 34 for buffer control, *n* = 25 for BsEXLX1, and *n* = 15 to 19 for different PcaLOOL proteins. Asterisks indicate statistical significance (**, *P* < 1%; ***, *P* < 0.01%).

**(ii) Weakening of filter paper.** Three of the four wild-type PcaLOOLs weakened cellulose filter paper, as measured by using the breaking force assay (39–41) (Fig. 3). In this assay, filter paper strips are incubated in buffered solutions containing the target protein and then clamped in an extensometer that records the force required to break the material (42). Compared to the buffer treatments, the application of PcaLOOL7, PcaLOOL9, and PcaLOOL12 significantly (*P* ≤ 0.05) decreased the breaking force of filter paper strips by 11 to 16%, compared to a 30% reduction with BsEXLX1 (Fig. 3). The low binding to Avicel and yet the ability to weaken cellulose filter paper suggest that the ability to desorb from cellulose surfaces is important to PcaLOOL function. Consistent with this interpretation, fusing the BsEXLX1 D2 domain (i.e., CBM63) to the C terminus of PcaLOOL2, PcaLOOL7, and PcaLOOL12 did not increase filter paper weakening by the proteins; moreover, the CBM63 fusion appeared to reduce the impact of PcaLOOLs (Fig. 3). Notably, previous mutagenesis of the D2 domain of BsEXLX1 revealed a nonlinear relation between microbial expansin binding to cellulose and filter paper weakening (43). Based on these results, further assessment of the PcaLOOLs on cellulosic materials focused on the wild-type proteins.

**(iii) Impact of PcaLOOLs on cell wall creep.** The defining characteristic of plant expansins and microbial expansin-like proteins is their ability to induce time-dependent, irreversible elongation (creep) of plant cell walls. The corresponding plant cell wall creep assay is the most diagnostic, specific, and sensitive method to detect expansin activity (12, 17). While the induction of cell wall creep correlates well with weakening of filter paper in BsEXLX1 and its mutants, this connection is much weaker for other microbial expansin-related proteins (17, 39). Indeed, none of the PcaLOOLs investigated herein were able to increase the extension rate of wheat coleoptile cell walls, a finding in accordance with the behavior of other single-domain DPBB proteins (10) (Fig. S6).

### Impact of PcaLOOLs on the viscoelastic behavior of CNF.

Rheology measurements were obtained to further quantify the impact of PcaLOOLs on cellulose fiber weakening. First, the linear viscoelastic (LVE) region was determined, and then time sweep, frequency sweep, and amplitude sweep measurements were performed. For all experiments using cellulose nanofibers (CNF), the storage (G′) and loss (G″) moduli did not change significantly over time (Supplemental File S1). The expected gel-like behavior of the starting CNF was further confirmed by the frequency sweep where G′ was invariably higher than G″ (Supplemental File S2) (44) and the amplitude sweep where G′ dropped below G″ at a high strain (i.e., at the limiting value of the LVE region) (Supplemental File S3). The limiting value of the LVE region specifies the yield stress of the material, which is the peak value of the elastic stress (σ′), calculated by the formula σ′ = G'γ, where G′ is the storage modulus and γ is the strain amplitude (45, 46). The corresponding strain amplitude at the yield stress is defined as the yield strain (Supplemental Files S4 and S5).

**FIG 4:**
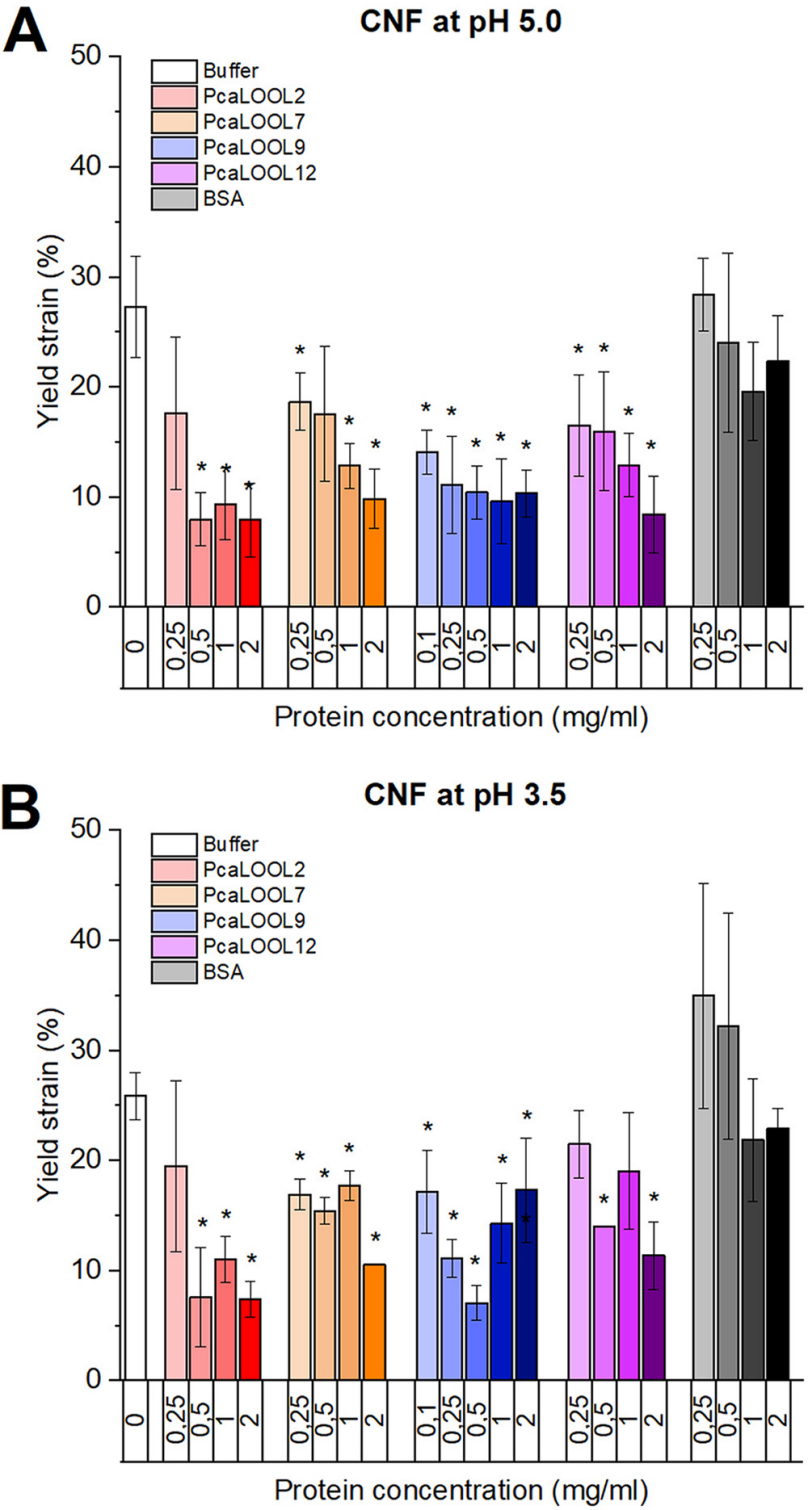
Yield strain determination, measured by means of oscillatory rheological tests. An 0.6% concentration of CNF (cellulose nanofibrils) was treated with increasing concentrations of protein for 24 h at room temperature and subjected to rheological tests. (A) CNF treated with PcaLOOLs at pH 5.0; (B) CNF treated with PcaLOOLs at pH 3.5. Asterisks indicate statistical significance (*P* ≤ 0.05; two-tailed *t* test) of decreased yield strain upon PcaLOOL treatment of CNF compared to that of buffer only. *n* = 2 to 5; error bars indicate standard deviations. Yield stress data are shown in Fig. S6 in the supplemental material. Raw data values are provided in Supplemental Files S4 and S5.

All PcaLOOLs lowered the yield strain of the CNF beyond negligible impacts observed using BSA (Fig. 4A and B; Fig. S7), consistent with PcaLOOL-mediated disruption of the fibrillar network. At pH 5.0, the impact of PcaLOOLs on the CNF network increased with increasing protein concentration (Fig. 4A). A decrease in yield strain of CNF was also observed after treatment with PcaLOOLs at pH 3.5; however, a clear correlation between yield strain and protein concentration was not observed at that pH value. Notably, at pH 3.5 and comparatively low protein concentrations, the BSA treatment increased the yield strain of CNF (Fig. 4B), which can be explained by the expected unfolding and resulting increase in surface charge previously reported for BSA under acidic conditions (47).

Considering that the pKa of Asp is 3.9, the putative “catalytic” Asp residue of PcaLOOLs should be largely protonated at pH 3.5 and deprotonated at pH 5.0. Accordingly, the present results suggest that PcaLOOLs function better at pH values at which the catalytic Asp is deprotonated. Similarly, cell wall creep by BsEXLX1 was highest above pH 5.5 (up to pH 9.5, where the protein begins to precipitate) (17).

### Impact of PcaLOOLs on cellulase action.

Each of the PcaLOOLs produced herein was tested for the ability to boost Cellic CTec2, Celluclast, or *endo*-1,4-β-d-glucanase action on Avicel PH-101 and Whatman qualitative filter paper. Notably, despite their ability to weaken filter paper and CNF networks, none of the PcaLOOLs substantially increased cellulase action on selected cellulosic substrates (Fig. S8 and S9). Indeed, reported impacts on biomass deconstruction differ considerably and depend strongly on the biomass source (26, 27, 48). For example, previous experiments showing complementary action between LOOS1 and a commercial cellulase were performed using cotton fiber and Agave tequilana fibers (19). Since PcaLOOL2 and PcaLOOL12 were recently shown to boost xylanase migration through cellulose-xylan composites (49), future studies of PcaLOOL impacts on the enzymatic deconstruction of cellulosic substrates should include complex lignocellulosic materials from diverse biomass sources.

### Activity of PcaLOOLs on chitinous substrates.

**(i) Binding to chitin.** Previous coexpression analyses clustered PcaLOOL2 with a predicted carbohydrate esterase family 9 *N*-acetylglucosamine 6-phosphate deacetylase, PcaLOOL7 with a putative glycoside hydrolase family 18 chitinase, and PcaLOOL12 with a predicted glycosyl transferase family 2 chitin synthase (28). These earlier observations point to a possible role for PcaLOOLs in fungal cell wall morphogenesis, potentially by targeting chitin in fungal cell walls during hyphal growth. To assess this possibility, investigations that evaluate the impacts of PcaLOOLs on chitinous substrates were initiated.

A clear pH dependency was observed for PcaLOOL binding to chitin, where after 1 h at room temperature, the highest binding in all cases was measured between pH 5.0 and 6.0 (Fig. 2B). At these pH values, the highest binding was observed for PcaLOOL7 and then by PcaLOOL12, which belong to different loosenin subgroups. Consistent with the reported cellulose binding activity of the CBM63 D2 domain of BsEXLX1, fusing the CBM63 to the PcaLOOLs did not substantially increase protein binding to chitin at pH 5.0. An increase in PcaLOOL binding to chitin, however, was observed at pH 6.0 and 7.0, where the CBM63 domain alone also showed the highest binding toward chitin. Despite the clear binding of wild-type PcaLOOLs to chitin, none substantially boosted chitinase action on commercial alpha-chitin (Fig. S10).

**(ii) Impact of PcaLOOLs on fungal growth.** The cell walls of most filamentous fungi comprise interwoven microfibrils of chitin and β-1,3-glucans that are embedded in α-1,3-glucans and glycoproteins (50). Accordingly, the comparatively high binding of PcaLOOLs to chitin motivated preliminary experiments that investigated the impacts of PcaLOOLs on mycelial growth. Since there is no readily available genetic system for manipulating *P. carnosa*, the impact of the PcaLOOLs analyzed herein on the phenotype of filamentous fungi was assessed by external protein supplementation to fungal cultivations. Given the particularly high binding of PcaLOOL7 and PcaLOOL12 to chitin, these proteins were selected for the fungal growth study; PcaLOOL2 was also included as a representative LOOL from subgroup A that had an N-terminal extension.

**FIG 5:**
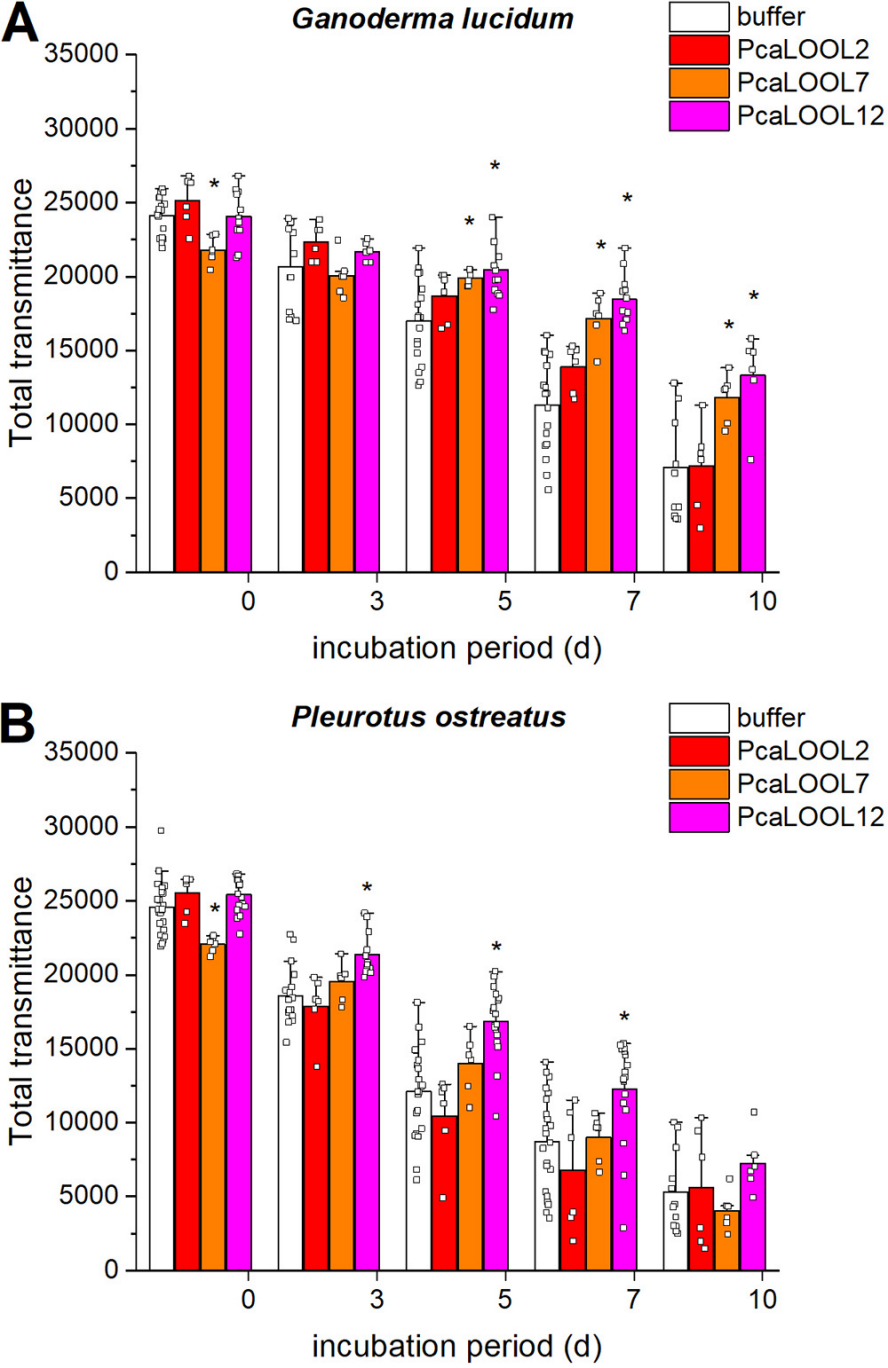
Effect of PcaLOOLs on the transmission of *G. lucidum* and *P. ostreatus* cultures. Malt liquid (2 mL) was inoculated with fungal mycelium and grown at 21°C for up to 10 days with 0.1 mg/mL PcaLOOL. Fungal growth was monitored at 880 nm; *n* ≥ 6, error bars indicate standard deviations. Asterisks indicate statistical significance (*P ≤ *0.05; two-tailed *t* test) of increased total transmission upon PcaLOOL supplementation compared to that of buffer only supplementation. (A) Total transmission of *G. lucidum* cultures containing PcaLOOL2 (red), PcaLOOL7 (orange), or PcaLOOL12 (magenta). (B) Total transmission of *P. ostreatus* cultures containing PcaLOOL2 (red), PcaLOOL7 (orange), or PcaLOOL12 (magenta).

Like *P. carnosa*, Ganoderma lucidum is a white-rot basidiomycete belonging to the Agaricomycetes class. *G. lucidum* is also one of the most common fungal species used for the production of mycelium composite materials (51, 52). BLAST searches of available *G. lucidum* sequences did not detect significant similarities to PcaLOOL7, PcaLOOL2, or PcaLOOL12, which reduced the possibility that native proteins would mask the impact of the supplemented PcaLOOLs. *G. lucidum* was thus grown in liquid malt extract supplemented with 0.1 mg/mL protein, and mycelial growth was followed over 10 days by light scattering (signal over sample height). In comparison to the transmission of *G. lucidum* reference cultures, the transmission of *G. lucidum* cultures amended with PcaLOOL7 and PcaLOOL12 increased between days 5 and 10 (Fig. 5A). The impact of PcaLOOLs on fungal growth was similar in cultivations of the white-rot Pleurotus ostreatus (Fig. 5B). Since no measurable difference in fungal growth rate was observed in *G. lucidum* and *P. ostreatus* cultivations amended with PcaLOOLs, it appears that differences in the transmission of cultures amended with PcaLOOL12, and to a lesser extent PcaLOOL7, result from changes to the density of cultivated mycelia.

### Conclusions.

The direct comparison of four loosenin-like proteins from a single filamentous fungus, *Phanerochaete carnosa*, uncovered functional characteristics that were common to all selected proteins and thereby deepen our understanding of microbial expansin-related proteins that comprise only the D1 (GH45-like) domain. In particular, all bound more strongly to alpha-chitin than to selected celluloses, and none of the PcaLOOLs displayed lytic activity. Despite only weak binding to cellulose, three of the four PcaLOOLs induced cellulose filter paper weakening and all of the PcaLOOLs reduced the yield strain of cellulose nanofibril networks. Moreover, increasing PcaLOOL binding to cellulose through fusion to the CBM63 domain of BsEXLX1 did not translate to an increase in cellulose filter paper weakening. The lack of synergistic action between PcaLOOLs and cellulolytic enzymes could reflect the comparative purity of the cellulose substrates used and motivates future studies that include complex lignocellulosic substrates that retain so-called biomechanical hot spots potentially targeted by expansin-related proteins. Especially intriguing was the observed correlation between PcaLOOL binding to chitin and the impact on cultivated fungal mycelia. Similar to the impact of plant expansins on plant cell wall formation, and of certain bacterial expansin-like proteins on bacterial cell wall formation, it is conceivable that fungal loosenins and other fungal expansin-related proteins play a role in fungal cell wall morphogenesis. This compelling possibility provides new direction to fundamental studies that investigate the biological role of microbial expansin-related proteins and opens new application concepts for their use in fungal biomass processing.

## MATERIALS AND METHODS

### Substrates and chemicals.

Cellulose nanofibers (CNF) were prepared from never-dried bleached kraft birch pulp by fluidization in deionized water six times (six passes) using a Voith LR40 homogenizer, as described by Österberg et al. (53). The resulting CNF samples with a solid content of 2.8% (wt/vol) were stored at 4°C. Avicel PH-101 (~50-μm particle size; catalog no. 11365), xylan from birch wood (catalog no. 95588), Whatman qualitative filter paper grade 1 (catalog no. WHA1001185), Whatman qualitative filter paper grade 3 (catalog no. WHA1003185), and peptidoglycan from Micrococcus luteus (catalog no. 53243) were purchased from Sigma-Aldrich (St. Louis, MO, USA). Chitin from shrimp shells (catalog no. *P*-CHITN), glucomannan (Konjac; low viscosity; catalog no. *P*-GLCML), carboxymethyl cellulose 4M (CMC; catalog no. *P*-CMC4M), xyloglucan (tamarind; catalog no. *P*-XYGLN), hexaacetyl-chitohexaose (catalog no. O-CHI6), cellohexaose (catalog no. O-CHE), and β-glucan (yeast; alkali soluble; catalog no. *P*-BGYST) were obtained from Megazyme (Bray, Ireland). Thermo Fisher Scientific (Waltham, MA, USA) was the supplier of the Pierce bicinchoninic acid (BCA) protein assay kit (catalog no. 23225). All other chemicals were reagent grade.

### Enzymes and reference microbial expansin-like protein.

The cellulase mixture Cellic CTec2 (cellulase, enzyme blend; catalog no. SAE0020), Celluclast (cellulase from Trichoderma reesei; catalog no. C2730), chitinase from *Trichoderma viride* (catalog no. C8241), lysozyme (from hen egg white; catalog no. 10837059001), and α-chymotrypsin (from bovine pancreas; catalog no. C6423) were obtained from Sigma-Aldrich, PNGase F was from New England Biolabs (Ipswich, MA, USA; catalog no. P0705S), and *endo*-1,4-β-d-glucanase (cellulase from *Trichoderma longibrachiatum*, glycoside hydrolase family 7; catalog no. E-CELTR) was from Megazyme. BsEXLX1 (expansin from Bacillus subtilis) was recombinantly expressed in Escherichia coli BL21 and purified as described by Georgelis et al. (17).

### Sequence analysis.

Amino acid sequences of loosenin-like proteins PcaLOOL2 (GenBank accession no. EKM55357.1), PcaLOOL7 (GenBank accession no. EKM53490.1), PcaLOOL9 (GenBank accession no. EKM52742.1), and PcaLOOL12 (GenBank accession no. EKM51974.1) from *P. carnosa* (28) were retrieved from the NCBI protein database (http://www.ncbi.nlm.nih.gov/protein/). Secretion signal prediction was performed using the SignalP v.4.1 web server (54). Amino acid sequences were aligned with Clustal Omega (55), and sequence similarities were visualized using the program ESPript 3 (http://espript.ibcp.fr) (56). Expasy tools ProtParam (57), Prosite (58), and Colab AlphaFold2 (59) were used to compute physicochemical protein parameters; DISULFIND (60) was used to predict disulfide bridges. Briefly, Colab AlphaFold2 was used to construct monomeric and homo-oligomeric structural models of the recombinant PcaLOOLs, where mmseqs2 was used for the multiple sequence alignment and the max_recycles value was set to 48. Models receiving the highest score were submitted to https://server.poissonboltzmann.org to prepare structures for continuum solvation calculations performed using the Adaptive Poisson-Boltzmann Solver method (61). The prediction of N- and O-glycosylation sites was carried out using the NetNGlyc 1.0 server (62), the NetOGlyc 4.0 server (63), and the GPP Prediction Server (64) and then visualized using PyMOL 2.3.

### PcaLOOL production and purification.

Selected PcaLOOLs were expressed in Pichia pastoris strain SMD1168H in accordance with the manufacturer’s instructions (Invitrogen, Thermo Fisher Scientific). Briefly, codon-optimized genes encoding each PcaLOOL were obtained as subcloned in pPICZαA plasmids with a C-terminal 6×His tag (GenScript, Piscataway, NJ, USA). P. pastoris transformants were screened for protein expression by immunocolony blotting using nitrocellulose membranes (Bio-Rad Laboratories, Hercules, CA, USA; catalog no. 1620115), Tetra·His antibodies (Qiagen, Hilden, Germany; catalog no. 34670), anti-mouse IgG (whole molecule), peroxidase antibodies produced in rabbit (Sigma-Aldrich; catalog no. A9044), and SuperSignal West Pico Plus chemiluminescent substrate (Thermo Fisher Scientific; catalog no. 34579). Precultures (4 × 50 mL in 500-mL baffled flasks) of the best transformant for each PcaLOOL were then grown in buffered glycerol-complex medium (BMGY; 100 mM potassium phosphate buffer [pH 6.0], 2% [wt/vol] peptone, 1% [wt/vol] yeast extract, 1.34% [wt/vol] yeast nitrogen base, 4 × 10^−5^% [wt/vol] biotin, 1% [vol/vol] glycerol) at 30°C and 100 rpm until an optical density at 600 nm (OD_600_) of ~6 was reached. Cells were harvested by centrifugation at 15°C and 1,500 × *g* for 10 min, and the obtained pellets were suspended in 4 × 300 mL of methanol-complex medium (BMMY) containing 0.5% (vol/vol) methanol instead of glycerol. Each 300-mL cultivation was performed in a 2.5-L Tunair shake flask covered with two layers of sterile Miracloth. Methanol was added to 1% (vol/vol) every 12 h, and induction was continued over 132 h at 28°C and 130 rpm. After the induction, culture supernatants were recovered and filtered, and the secreted recombinant proteins were purified by affinity chromatography using Ni-NTA resin (Qiagen; catalog no. 30230). Specifically, supernatants were concentrated using a Vivaflow 200 crossflow cassette with a 10,000-molecular-weight-cutoff (MWCO) polyethersulphone (PES) membrane (Sartorius, Göttingen, Germany; catalog no. VF20P0) and loaded onto a 5-mL GE Healthcare HisTrap FF crude prepacked column (Thermo Fisher Scientific; catalog no. 11723219). Proteins were eluted by fast protein liquid chromatography (FPLC) using an ÄKTA purifier (Amersham BioScience, Amersham, UK).

The purified proteins were concentrated and transferred to 10 mM sodium citrate buffer (pH 5.0) using Vivaspin Turbo 4 ultrafiltration units with a 5,000-MWCO PES membrane (Sartorius; catalog no. VS04T11). The purity and concentration of each PcaLOOL were assessed by SDS-PAGE and the Pierce BCA protein assay kit, respectively, prior to storage at −80°C. To confirm protein identity, deglycosylated forms of the purified PcaLOOLs were prepared using peptide-*N*-glycosidase F (PNGase) prior to digestion with α-chymotrypsin and analysis using an ultrafleXtreme MALDI- TOF/TOF mass spectrometer (Bruker, Billerica, MA, USA).

### Construction of PcaLOOL-CBM63 fusions.

PcaLOOL2, PcaLOOL7, and PcaLOOL12 were selected to design three separate fusion proteins that comprise at the C terminus the CBM63 from expansin-like protein EXLX1 encoded by Bacillus subtilis (accession no. WP_014664195) (15). In all cases, the linker sequence connecting the two domains of each fusion protein was taken from the EXLX1 protein and included the two last amino acids from EXLX1 domain 1 (amino acid sequence of the linker in fusion proteins, KAPITG). The genes encoding the resulting fusion proteins, PcaLOOL2-CBM63, PcaLOOL7-CBM63, and PcaLOOL12-CBM63, were codon optimized for expression in Pichia pastoris and separately cloned in the pPICZαA plasmid, which imparts a C-terminal His_6_ tag (GenScript).

The expression plasmids were transformed to P. pastoris strain SMD1168H by electroporation. The resulting transformants were induced on BMMY and then screened for protein expression by immunocolony blotting as described above. For protein production, P. pastoris transformants expressing PcaLOOL2-CBM63 and PcaLOOL7-CBM63 were grown overnight in 50 mL of BMGY medium at 30°C with continuous shaking at 220 rpm. The cells were harvested by centrifugation and suspended in 350 mL of BMMY medium in 2.5-L Tunair shake flasks to an OD_600_ of ~0.9. Cultures were grown at 20°C and 140 rpm for 5.5 days, and 1% methanol was added every 24 h to induce recombinant protein expression. PcaLOOL2-CBM63 and PcaLOOL7-CBM63 were purified from the culture supernatants by affinity chromatography as described above and then stored in 10 mM sodium citrate (pH 5.0) at −80°C. PcaLOOL12-CBM63 was produced as described above albeit at smaller scale (i.e., 100 mL of BMMY medium in a 500-mL nonbaffled Erlenmeyer flask) and then purified by batch purification. In this case, the pH of the culture supernatant was adjusted to 7.8 with NaOH and filtered with a 0.45-μm PES membrane, after which 12 mL of Ni-NTA agarose resin was suspended in the pH-adjusted culture supernatant for 16 h at 5 to 10°C. The resin was then transferred into two empty gravity flow columns (Bio-Rad, Econo-Pac) and washed with 10 column volumes of 50 mM Tris-HCl (pH 7.8) containing 150 mM NaCl and 10 mM imidazole, and then the bound protein was eluted using 4 column volumes of 20% elution buffer, 4 column volumes of 50% elution buffer, and 5 column volumes of 100% elution buffer (50 mM Tris-HCl [pH 7.8] containing 150 mM NaCl and 500 mM imidazole). After purification, the protein was transferred to 10 mM sodium acetate (pH 5.0) using a 10-kDa Vivaspin Turbo 4. The CBM63 domain comprising a C-terminal His_6_ tag was produced in Escherichia coli using the BL21(DE3) strain and pET21a(+) plasmid. In this case, E. coli transformants were grown in 500 mL of Luria-Bertani (LB) medium in 2-L nonbaffled glass Erlenmeyer flasks at 37°C with shaking at 200 rpm; when the cultures reached an OD_600_ of 0.6, the cultivation temperature was reduced to 20°C and 0.5 mM IPTG (isopropyl-β-d-thiogalactopyranoside) was added to induce recombinant protein expression. Cells were harvested after 16 h and lysed by sonication, and the cell lysate was clarified by centrifugation and filtration using a 0.45-μm PES membrane before batch purification of the CBM63 domain using Ni-NTA agarose resin. Purified protein was transferred to 4 to 10 mM sodium acetate, pH 5.0, using a 5-kDa Vivaspin Turbo.

### CD spectroscopy.

The recombinant PcaLOOLs were diluted to 0.1 mg/mL in H_2_O. Circular dichroism (CD) spectroscopy was performed using a Chirascan CD spectrometer (Applied Photophysics, Leatherhead, UK). CD data were collected between 190 and 280 nm at 22°C using a 0.1-cm-path-length quartz cuvette. CD measurements were acquired every 1 nm with 0.5 s as an integration time and repeated three times with baseline correction. A Chirascan Pro-Data Viewer (Applied Photophysics) was used to convert direct CD measurements (θ; mdeg) to mean residue molar ellipticity ([θ]MR), and secondary structures were predicted using the BeStSel web server (65) from 190 to 250 nm and a scale factor of 1. Thermal unfolding was recorded from 20°C to 80°C between 190 and 280 nm with a 2°C step size at a 1°C/min ramp rate with ±0.2°C tolerance. The melting temperature was analyzed with Global3 (Applied Photophysics).

### Test for hydrolytic activity.

Purified proteins were tested for hydrolytic activities toward xylan from birchwood, CMC, and glucomannan. Substrates were suspended in 50 mM sodium acetate buffer (pH 5.0) to a final concentration of 1% (wt/vol); 125 μL of each substrate was then transferred to separate wells in a 96-well plate (Thermo Fisher Scientific) and supplemented with PcaLOOLs (final concentration, 0.01 mg/mL) and Milli-Q water to a final sample volume of 250 μL. BSA was used as a reference. Plates were incubated for 16 h in a ThermoMixer C set at 40°C and 700 rpm. The reducing sugar concentration was determined by the *para*-hydroxybenzoic acid hydrazide (PAHBAH) assay calibrated against glucose (66).

### Test for lytic activity.

For the turbidimetric assay, water-insoluble substrates (β-glucan from yeast, peptidoglycan from Micrococcus luteus) were suspended at 0.35 mg/mL in 50 mM sodium acetate buffer (pH 5.0) and supplemented with PcaLOOL or BSA to a final protein concentration of 0.05 mg/mL. Reaction mixtures were incubated in a ThermoMixer C set at 1,000 rpm and 25°C for 0 to 24 h. At regular intervals, 0.12-mL samples were collected and analyzed spectrophotometrically at 600 nm to detect substrate solubilization. Samples without protein served as negative controls.

For the analysis of reaction samples by thin-layer chromatography (TLC), polysaccharide substrates (xyloglycan, peptidoglycan, β-glucan) were suspended at 0.35 mg/mL in 50 mM sodium acetate buffer (pH 5.0) and supplemented with PcaLOOL to a final protein concentration of 0.05 mg/mL. Oligosaccharide substrates (cellohexaose, hexaacetyl-chitohexaose) were suspended at 1 mM in 50 mM sodium acetate buffer (pH 5.0) and mixed with 0.125 mg/mL PcaLOOL. Reaction samples were incubated as described above for a period of 24 h. BSA was used as a reference on polysaccharide and oligosaccharide substrates; lysozyme and *endo*-1,4-β-d-glucanase were also included as reference treatments of oligosaccharide substrates. Samples (2 μL of polysaccharide reaction samples, 10 μL of oligosaccharide reaction samples) and standards (1 μL of 250 mM glucose, 250 mM xylose, or 225 mM *N*-acetylglucosamine) were applied to a TLC silica gel 60 F254 (Sigma-Aldrich). Chromatograms were developed in an *n*-propanol–25% ammonia (2:1) solvent mixture as the eluent. Spots were visualized by spraying with 10% sulfuric acid in ethanol, followed by heating of the plates using a Steinel HL 1920E hot air blower.

### Binding studies.

Protein adsorption to cellulose (Avicel PH-101) or chitin from shrimp shells was monitored by a pulldown assay. Specifically, 12.5 mg (to achieve 2.5% [wt/vol]) of substrate was weighed into 1.5-mL Eppendorf LoBind microcentrifuge tubes (Sigma-Aldrich) and suspended in a final reaction volume of 500 μL of 50 mM buffer supplemented with 0.1 mg/mL target protein. Buffers included 50 mM sodium citrate (pH 3.5), 50 mM sodium acetate buffer (pH 5.0), 50 mM sodium phosphate (pH 6.0), and 50 mM sodium phosphate (pH 7.0). BSA (Sigma-Aldrich; catalog no. A3059) was used as a reference. Protein blanks were prepared using protein solutions without substrate; substrate blanks contained substrate suspensions without protein. Samples were prepared in triplicate and incubated for 1 h at room temperature on a tube rotator set to 20 rpm; supernatants were then recovered by centrifugation (15,000 rpm for 10 min), and protein concentrations were measured using the Pierce BCA protein assay kit.

### Paper weakening assay.

The ability to weaken filter paper was analyzed as described by Cosgrove et al. (42). Briefly, Whatman qualitative filter paper grade 3 was cut into 10- by 2.0-mm strips and soaked in 1 mL of 20 mM MES (morpholineethanesulfonic acid) buffer (pH 6.0) containing 0.2 mg/mL protein. BsEXLX1 was used as a positive reference. Incubation was performed with gentle inversion to equilibrate the solution with the strips at 25°C for 4 h. After incubation, the filter paper strips were fixed between two clamps of a custom-built extensometer (42) and extended at 1.5 mm/min while the tensile force on a digital chart recorder was recorded. The maximum force attained was taken as the breaking force.

### Cell wall extension (creep) assay.

Etiolated wheat (Triticum aestivum L. cv Pennmore) coleoptiles were prepared as described by Cosgrove et al. (42), heat inactivated by a 15-s dip in boiling water, and fixed between two clamps of a custom-built constant-force extensometer for cell wall creep experiments (1), which resulted in a tensile force of 20 *g* on the specimen. The specimen was kept in 200 μL of 20 mM MES buffer (pH 6.0) for 10 min, and then the buffer was replaced with 50 mM NaOH to increase the sensitivity of the material in the creep assay. After 10 min, the coleoptiles were rinsed in 20 mM MES buffer (pH 6.0), and after an additional 15 min, the buffer was replaced with fresh buffer containing 0.2 mg/mL protein.

### Rheological measurements.

Rheological measurements were performed using CNF. The stock solution (2.8% [wt/vol]) was first dispersed in 0.423 mL H_2_O and sonicated with a tip sonicator (Q500; QSonica, Newton, CT, USA) at 20% amplitude for 2 min (2-s on/off cycles). The final concentrations of the sample components were 0.6% (wt/vol) substrate, 5 mM sodium acetate buffer (pH 5.0 and 3.5), and between 0.1 and 2 mg/mL of protein; the concentration of 0.6% (wt/vol) CNF was chosen based on previous research (67). All sample mixtures (0.5 mL) were incubated for 24 h at room temperature prior to the transfer of 70 μL to a smooth 8-mm plate with a 1-mm measuring gap for rheometry measurements using an Anton Paar Physica MCR 302 rheometer (Anton Paar, Graz, Austria). The temperature (23°C) was controlled with an H-PTD 200 Peltier hood (Anton Paar). To counter evaporation effects, the Peltier hood and a filled water ring were attached. The oscillatory tests consisted of three successive intervals: time sweep, frequency sweep, and amplitude sweep. Time sweeps were performed at a constant strain amplitude of 1% and an angular frequency of 1 s^−1^, frequency sweeps were performed at angular frequencies of 100 to 1 s^−1^ (at constant strain amplitude 1%), and amplitude sweeps were performed in the strain amplitude range from 0.1% to 100% (at a constant angular frequency of 1 s^−1^). The time and frequency sweeps were performed at the linear viscoelastic region. The mechanical spectra for storage modulus (G′) and loss modulus (G′′) were recorded to determine the viscoelastic properties of the CNF dispersions.

### Assay for synergism in enzymatic polysaccharide hydrolysis.

Synergism studies were performed in 2-mL Eppendorf tubes in a total reaction volume of 1 mL. Tested cellulose substrates (Avicel PH-101 or Whatman qualitative filter paper grade 1 disks created using a hole punch) were suspended in 50 mM sodium acetate buffer (pH 5.0) to a final concentration of 25 mg/mL. Substrate suspensions were then treated in two ways: (i) treatment with a mixture of a given PcaLOOL (0.041 mg/mL) and the commercial cellulase Cellic CTec2 (0.405 mg/mL) for 24 h in a ThermoMixer C set at 40°C and 1,000 rpm or (ii) pretreatment with a given PcaLOOL (0.041 mg/mL) for 1 h in a ThermoMixer C set at 25°C and 1,000 rpm prior to addition of Cellic CTec2 (0.405 mg/mL), after which the incubation proceeded for 24 h at 40°C and 1,000 rpm. BSA was used as a reference. Aliquots were sampled at regular time intervals and analyzed by the *para*-hydroxybenzoic acid hydrazide (PAHBAH) assay. Substrate suspensions of Whatman qualitative filter paper grade 1 were treated in three additional ways: (i) treatment with a mixture of a given PcaLOOL (0.7 mg/mL) and the commercial cellulase Celluclast (7 mg/mL) for 24 h in a ThermoMixer C set at 50°C and 1,000 rpm, (ii) treatment with a mixture of a given PcaLOOL (0.05 mg/mL) and a commercial *endo*-1,4-β-d-glucanase (0.5 mg/mL) for 24 h in a ThermoMixer C set at 50°C and 1,000 rpm, or (iii) pretreatment with a given PcaLOOL (0.041 mg/mL) for 72 h in a ThermoMixer C set at 25°C and 1,000 rpm prior to the addition of Cellic CTec2 (0.405 mg/mL), after which the incubation proceeded for 2 h at 40°C and 1,000 rpm. Aliquots were sampled at the end of the incubation period and analyzed by the 3,5-dinitrosalicylic acid (DNS) assay calibrated against glucose (68).

Synergism experiments were also performed using chitin from shrimp shells and a commercial chitinase from *T. viride*. Chitin was suspended in 50 mM sodium acetate buffer (pH 6.0) to a final concentration of 25 mg/mL and treated in two ways: (i) treatment with a mixture of a given PcaLOOL (0.008 mg/mL) and chitinase (0.08 mg/mL) for 24 h in a ThermoMixer C set at 25°C and 1,000 rpm or (ii) pretreatment with a given PcaLOOL (0.75 mg/mL) for 24 h in a ThermoMixer C set at 25°C and 1,000 rpm prior to addition of chitinase (0.02 mg/mL), after which the incubation proceeded for another 24 h at the same conditions. BSA was used as a reference, and aliquots sampled after 24 h were analyzed by the DNS assay calibrated against glucose.

### Mycelial growth experiments.

Ganoderma lucidum (reishi mushroom; HAMBI FBCC665) and Pleurotus ostreatus (oyster mushroom; HAMBI FBCC0515) were obtained from the HAMBI Culture Collection (University of Helsinki, Faculty of Agriculture and Forestry, Department of Microbiology). The isolated fungal cultures were previously identified by internal transcribed spacer PCR (ITS-PCR) (51). Fungal cultures were propagated and maintained on 2% (wt/wt) malt extract (LabM, Heywood, UK) in 2% (wt/wt) agar (Scharlab, Sentmenat, Spain) at 4°C ± 1°C. For mycelial growth experiments, 2 mL of malt extract (2% [wt/wt]) in 4-mL screw neck vials (Fisher Scientific, Loughborough, UK) were inoculated with an agar piece of the *G. lucidum* or *P. ostreatus* culture. Malt extract was then supplemented with 0.1 mg/mL of PcaLOOL2, PcaLOOL7, or PcaLOOL12 prepared in 50 mM sodium acetate buffer (pH 5.0). Vials were sealed with cotton filters and open top screw caps (La-Pha-Pak, Langerwehe, Germany). Static incubation of each culture was performed at 21°C (±1°C), and fungal growth was measured over 10 days by multiple light scattering using a Turbiscan LAB and adapter for a 4-mL vial (Formulaction, Toulouse, France). Transmission at 880 nm was recorded by a synchronous optical sensor that moves along the vertical axis at 40-μm intervals along the cylindrical measurement cell (69).
